# Frequency of Severe Malaria and Invasive Bacterial Infections among Children Admitted to a Rural Hospital in Burkina Faso

**DOI:** 10.1371/journal.pone.0089103

**Published:** 2014-02-14

**Authors:** Jessica Maltha, Issa Guiraud, Bérenger Kaboré, Palpouguini Lompo, Benedikt Ley, Emmanuel Bottieau, Chris Van Geet, Halidou Tinto, Jan Jacobs

**Affiliations:** 1 Department of Clinical Sciences, Institute of Tropical Medicine, Antwerp, Belgium; 2 Center for Molecular and Vascular Biology, University of Leuven, Leuven, Belgium; 3 IRSS / Clinical Research Unit of Nanoro (CRUN), Nanoro, Burkina Faso; 4 Pediatrics, University of Leuven, Leuven, Belgium; Pennsylvania State University College of Medicine, United States of America

## Abstract

**Background:**

Although severe malaria is an important cause of mortality among children in Burkina Faso, data on community-acquired invasive bacterial infections (IBI, bacteremia and meningitis) are lacking, as well as data on the involved pathogens and their antibiotic resistance rates.

**Methods:**

The present study was conducted in a rural hospital and health center in Burkina Faso, in a seasonal malaria transmission area. Hospitalized children (<15 years) presenting with T≥38.0°C and/or signs of severe illness were enrolled upon admission. Malaria diagnosis and blood culture were performed for all participants, lumbar puncture when clinically indicated. We assessed the frequency of severe malaria (microscopically confirmed, according to World Health Organization definitions) and IBI, and the species distribution and antibiotic resistance of the bacterial pathogens causing IBI.

**Results:**

From July 2012 to July 2013, a total of 711 patients were included. Severe malaria was diagnosed in 292 (41.1%) children, including 8 (2.7%) with IBI co-infection. IBI was demonstrated in 67 (9.7%) children (bacteremia, n = 63; meningitis, n = 6), 8 (11.8%) were co-infected with malaria. Non-Typhoid *Salmonella* spp. (NTS) was the predominant isolate from blood culture (32.8%), followed by *Salmonella* Typhi (18.8%), *Streptococcus pneumoniae* (18.8%) and *Escherichia coli* (12.5%). High antibiotic resistance rates to first line antibiotics were observed, particularly among Gram-negative pathogens. In addition, decreased ciprofloxacin susceptibility and extended-spectrum beta lactamase (ESBL) production was reported for one NTS isolate each. ESBL production was observed in 3/8 *E. coli* isolates. In-hospital mortality was 8.2% and case-fatality rates for IBI (23.4%) were significantly higher compared to severe malaria (6.8%, *p*<0.001).

**Conclusions:**

Although severe malaria was the main cause of illness, IBI were not uncommon and had higher case-fatality rates. The high frequency, antibiotic resistance rates and mortality rates of community acquired IBI require improvement in hygiene, better diagnostic methods and revision of current treatment guidelines.

## Introduction

Malaria remains a major cause of mortality in children in most sub-Saharan African countries, despite decreasing incidence rates [1]. Community-acquired invasive bacterial infections (IBI) such as bacteremia and bacterial meningitis also contribute to the under-5 mortality in sub-Saharan Africa [2], [3], although exact data are lacking in many areas. Severe malaria and IBI present with similar symptoms [4], [5] precluding differential diagnosis on clinical grounds and leading to an overdiagnosis of malaria [6] and ignorance of IBI. In Burkina Faso, a West African country with seasonal malaria transmission, malaria is reported to be responsible for 55% of deaths at district level [7], but data on frequency of IBI, pathogens and susceptibility patterns are hardly available due to a lack of diagnostic microbiology facilities. We conducted a prospective observational study in order to assess proportions of severe malaria and community-acquired IBI among children admitted to a rural hospital and health center in Burkina Faso. In addition, we determined the causative bacteria and their antibiotic resistance patterns and assessed case-fatality rates of both IBI and severe malaria.

## Methods

### Study site

The survey was conducted at the district hospital Centre Médical avec Antenne chirurgicale (CMA) Saint Camille of Nanoro and the health center Urbain (with inpatient beds), both situated in Nanoro, Burkina Faso. Nanoro is located in a rural area in the Center-West region of the country, approximately 90 km west of the capital Ouagadougou. The health district of Nanoro comprises approximately 153,259 inhabitants of whom 35,159 (22.9%) are children <5 years of age [8]. In Nanoro there is hyperendemic malaria transmission from July – November, corresponding to the rainy season (June – October). Meningitis epidemics may occur in the hot, dry season (February – April), the last dating from 2009, after which a mass vaccination campaign against *Neisseria meningitidis A* was performed [7]. Pneumococcal and meningococcal vaccines were not included in the national extended program of immunization (EPI) at the time of the study period. Vaccination against *Haemophilus influenzae* type b was introduced into EPI in January 2006 and the estimated coverage of immunization was 95.2% in 2011 [8]. In 2010, the overall under-5 mortality rate in Burkina Faso was 129/1000 life-births and 142/1000 life-births in the Center-West region [7]; HIV-prevalence was 1.2% among women 15–49 years and 0.76% among pregnant women [7].

### Study population and procedures

All admitted children <15 years of age presenting with axillary temperature ≥38.0°C or clinical signs of severe illness were enrolled, including respiratory distress, prostration, altered consciousness, convulsions, clinical jaundice, hypothermia, signs of shock or severe malnutrition (weight for height score <70% according to national guidelines or kwashiorkor) or with severe anemia (hemoglobin <5 g/dl). Medical history, including prior antibiotic (48 h) and antimalarial (2 weeks) treatment, physical examination and outcome of febrile episode (died, referred, discharged) were registered on a standardized form by trained study nurses. Venous blood samples for blood culture, malaria diagnosis, full blood count and blood glucose levels were collected from all participants by trained study staff using aseptic techniques. Lumbar puncture was performed in case of suspicion of cerebral malaria and/or bacterial meningitis, based on the decision of the attending health care worker. Decision for HIV testing or other additional testing was left to the discretion of attending health staff. Treatment decisions were made by the attending health staff according to national guidelines: quinine IV for severe malaria, ampicillin + gentamicin for neonatal infections and ceftriaxone for suspected sepsis or meningitis in older children. Laboratory results were provided instantly to guide treatment decisions.

### Laboratory procedures

All laboratory analysis were performed at the Clinical Research Unit of Nanoro (CRUN) which is located on the compound of CMA. Samples were submitted within 15 minutes after collection.

Thick and thin blood films were stained with 3% Giemsa solution (pH 7.2), examined for presence of *Plasmodium* species according to standard procedures [9] and results were expressed as asexual parasites per microliter using the patient's white blood cell (WBC) count. Every slide was read by two experienced microscopists, and in case of discrepant results (positive vs. negative, different *Plasmodium* species, difference in parasite density >Log10 or ratio >2 in case of parasite density ≤400/µl and >400/µl respectively) by a third experienced microscopist. A selection of slides (5%) was re-read by an expert microscopist whose results were considered conclusive.

The malaria rapid diagnostic test (RDT) recommended by the national malaria control program, SD Bioline Pf (Standard Diagnostics, Hagal-Dong, Korea) detecting *P. falciparum*-specific histidine-rich protein-2 (PfHRP2), was performed on EDTA blood samples according to the manufacturers' instructions.

In all children, 1–3 ml of venous blood was collected into a pediatric blood culture bottle (BD BACTEC Peds Plus™/F, Becton Dickinson and Company, Sparks, Maryland, USA). A subset of blood culture bottles was weighed before and after blood inoculation to determine the amount of blood collected. All blood culture bottles were incubated in a BACTEC 9050 instrument (Becton Dickinson) for a total of 5 days. If flagged for growth they were Gram stained, subcultured on Eosin-Methylene blue (EMB) agar and 5% Sheep Blood agar (bioMérieux, Marcy-l'Etoile, France) and incubated at 35–37°C for 24 hours in atmospheric conditions and at 5% CO2 respectively. Isolates were identified to the species level by standard biochemical methods and antibiotic susceptibility testing was performed by disk diffusion according to CLSI criteria [10] or in case of assessment of minimal inhibitory concentration (MIC) values by E-test macromethod (bioMérieux) [10]. For *Salmonella* spp., multidrug resistance (MDR) was defined as resistance to the first-line antibiotics ampicillin, chloramphenicol and trimethoprim-sulfamethoxazole (TMP-SMX); reduced fluoroquinolone susceptibility (further referred to as decreased ciprofloxacin susceptibility, DCS) was defined as resistance to nalidixic acid [10] and/or MIC-values for ciprofloxacin >0.06 µg/ml [11]. *Enterobacteriaceae* resistant to cephalosporins were tested for Extended Spectrum Beta-Lactamase (ESBL) production by combined double-disk method (Rosco Diagnostica, Taastrup, Denmark) [10]. For E*nterobacteriaceae*, no breakpoints have been published for azithromycin susceptibility, however EUCAST V 3.1. [11] suggests treatment of infections with *Salmonella* Typhi at MIC-values ≤16 mg/l and recently this value has been proposed as an epidemiological cutoff value for wild-type *Salmonella* spp. [12]. Non-fragile clinically significant organisms were shipped on tryptic soy agar (TSA) at room temperature to the Institute of Tropical Medicine, Antwerp, where identification and antibiotic susceptibility testing were confirmed. *Shigella* spp. and a subset of *Salmonella* spp. isolates was confirmed at the National Reference Laboratory for *Salmonella* and *Shigella* (Institute of Public Health, Brussels) by slide agglutination with commercial monospecific antisera (Sifin, Berlin, Germany), following the Kauffmann-White scheme [13].

Cerebrospinal fluid (CSF) was assessed for its aspect, cell count, Gram stain and culture. The CSF leucocyte count was assessed in a Nageotte counting chamber at ×400 magnification, and if ≥50 WBC/µl were counted a thin film was prepared and stained with methylene blue to count the percentage of neutrophils and lymphocytes. For culture, 1 to 2 drops of CSF were inoculated onto blood agar and chocolate blood agar supplemented with Isovitalex™ (Becton Dickinson) and incubated at 35–37°C at 5% CO_2_ for 48 hours. Any bacterial growth observed was further processed by standard microbiological techniques. All CSF samples were assessed by latex agglutination testing for *Haemophilus influenzae b, Streptococcus pneumoniae, Streptococcus* group B, *Neisseria meningitidis* and *Escherichia coli* K1 (Pastorex™ Meningitis, BioRad, Marnes-la-Coquette, France). In addition if *N. meningitidis* was identified serotyping was performed (BioRad or Becton Dickinson).

Full blood counts were assessed using Sysmex XS1000i (Sysmex Corporation, Kobe, Japan).

For determination of glucose levels full blood was collected on NaF tubes and processed by Flexor Junior (Vital Scientific, Netherland). Screening for HIV was performed at the HIV center on the CMA compound according to national guidelines by the rapid diagnostic test Determine™ HIV-1/2 (Alere Medical Co., Ltd, Chiba, Japan) and in case positive the SD Bioline HIV-1/2 3.0 (Standard Diagnostic INC, Kyonggi-do, Korea) was performed.

### Case definitions

Fever was defined as axillary temperature ≥37.5°C or reported history of fever in the past 48 hours.

Malaria was defined as the presence of asexual *P. falciparum* parasites confirmed by microscopy.

Severe malaria was defined as microscopically confirmed malaria and fulfillment of at least one of the WHO clinical or laboratory criteria of severe malaria [14] with slight adaptations: respiratory distress was defined as abnormal deep breathing, subcostal retraction or tachypnea according to age [15]; shock was defined as an abnormal low systolic blood pressure according to age [15], or a combination of temperature gradient (warm trunk and cold extremities) and capillary refill >3 seconds.

Cerebral malaria was defined as severe *falciparum* malaria with coma (Glasgow coma scale <11 or Blantyre coma score <3) or convulsions in the past 24 hours (≥2 or postictal phase ≥30 minutes) and exclusion of bacterial meningitis or hypoglycemia alone (prompt recovery of consciousness after glucose infusion) as cause of symptoms [14].

IBI was defined as bacteremia and/or bacterial meningitis. Bacteremia was defined as the growth of clinical significant organisms from blood culture. Non-pathogenic bacteria or skin flora were considered contaminants, including coagulase-negative *Staphylococci*, *Bacillus* spp. and *Micrococcus* spp.

Bacterial meningitis was defined as i) cerebrospinal fluid (CSF) culture grown with an organism of known clinical significance, or ii) CSF white blood cell count >10/µl with on Gram stain slides the presence of organisms of known clinical significance, or iii) CSF white blood cell count >10/µl and blood culture grown with an organism of known causative role in meningitis or iv) positive latex agglutination. Probable meningitis was defined as a child with clinical signs of meningitis, in whom no lumbar puncture had been performed and a bacteria known to be a causative agent in meningitis was isolated from blood culture.

### Data management and analysis

Clinical and laboratory data was double entered in Epi Info version 3.5.3. Statistical analysis was done with Stata 11 (Stata Corp., College Station, TX, USA).

Categorical data were assessed for significance using the chi-square test or Fisher exact test as appropriate. The Wilcoxon rank sum test was used for non-parametric data. Case-fatality rates (CFR) were calculated as proportions of children who died within a specified disease group. Only participants for whom the final hospital outcome was known (discharge or death) were included in the denominator of CFR calculations, referred patients were excluded.

### Ethics statement

The study was conducted according to the principles expressed in the Declaration of Helsinki and was approved by the national ethics committee of Burkina Faso, the institutional review board of the Institute of Tropical Medicine, Antwerp and the ethics committee of the University Hospital of Antwerp. Written informed consent was given by all parents or guardians of children enrolled.

## Results

### Patients included

From July 2012 to July 2013, a total of 1134 children were admitted to the hospital, 337 were considered non-eligible and another 177 eligible children were not included for various reasons (not proceeding to study nurse, no blood sampling, refusal to provide informed consent). In total 711 admitted children were included: 607 at the hospital and another 104 children at the health center. For age, gender and outcome, there were no significant differences (*p*>0.05) between included and eligible non-included patients, however, recorded axillary temperature was significantly higher among included patients (*p*<0.001).

Demographic data, clinical features and laboratory results of the children included are displayed in Table 1. Children were included because of axillary temperature ≥38.0°C (n = 196), signs of severe illness with fever (n = 494) or without fever (n = 21). Lumbar puncture was performed in 19 children. Six children were known as HIV-positive at enrolment and an additional two (out of five tested) were newly diagnosed with HIV infection at inclusion.

**Table 1:** Demographic, clinical and laboratory characteristics upon admission.

	All (n = 711)	Hospital (n = 607)	Health center (n = 104)
Female sex, n (%)	318 (44.7)	264 (43.5)	54 (51.9)
Age, median months (IQR)	19 (10–36)	18 (9–34)	28.5 (14–65)
Axillary temperature ≥38.0°C	507 (71.3)	410 (67.5)	97 (93.3)
Pretreatment with antimalarials, n (%)	305 (42.9)	245 (40.4)	60 (57.7)
Pretreatment with antibiotics, n (%)	199 (28.0)	174 (28.7)	25 (24.0)
**Clinical features upon admission**			
Altered consciousness	33 (4.6)	28 (4.6)	5 (4.8)
Coma, n (%)	23 (3.2)	18 (3.0)	5 (4.8)
Convulsions, n (%)	55 (7.7)	44 (7.2)	11 (10.6)
Prostration, n (%)	304 (42.8)	262 (43.2)	42 (40.4)
Respiratory distress, n (%)	221 (31.1)	172 (28.3)	49 (47.1)
Shock	48 (6.8)	40 (6.6)	8 (7.7)
Jaundice, n (%)	30 (4.2)	30 (4.9)	0
Hemoglobinuria, n (%)	6 (0.8)	5 (0.8)	1 (1.0)
Severe malnutrition*, n (%)	85 (12.7)	80 (13.7)	5 (5.9)
**Laboratory features upon admission**			
Severe anemia (<5 g/dl), n (%)	191 (26.9)	186 (30.6)	5 (4.8)
Hypoglycemia (<2.2 mmol/l), n (%)	36 (5.1)	30 (4.9)	6 (5.8)
Hyperparasitemia (>250,000/µl)	22 (3.1)	16 (2.6)	6 (5.8)

Hb  =  hemoglobin IQR  =  interquartile range, *For 670 children data on severe malnutrition known.

### Severe malaria

In 378 children (53.2%) malaria was confirmed microscopically. In total 292 children (41.1%) fulfilled the diagnosis of severe malaria, of whom 48 children (16.4%) had cerebral malaria and 136 (46.6%) severe malaria anemia (Table 2), including 13 children who had both cerebral malaria and severe anemia. Severe malaria was more common among children aged 1–5 years than those <1 and >5 years (both *p*<0.001).

**Table 2:** Severe malaria and invasive bacterial infections by age group.

	All	<1 m	1–11 m	12–23 m	24–59 m	≥60 m
	n = 711	n = 15	n = 195	n = 206	n = 196	n = 99
Severe malaria*, n (%)	292 (41.1)		62 (31.8)	101 (49.0)	101 (51.5)	28 (28.3)
Cerebral malaria, n (%)	48 (6.8)		7 (3.6)	16 (7.8)	22 (11.2)	3 (3.0)
Severe anemia, n (%)	136 (19.1)		40 (20.5)	47 (22.8)	46 (23.5)	3 (3.0)
IBI^§^, n (%)	67 (9.2)	3 (20.0)	15 (7.7)	20 (9.7)	13 (6.6)	16 (16.2)
Bacteremia, n (%)	63 (8.9)	3 (20.0)	14 (7.2)	18 (8.7)	13 (6.6)	15 (15.2)
Confirmed + probable meningitis, n (%)	6+5 (1.5)	0+1 (6.7)	1+0 (0.5)	3+1 (1.9)	0+1 (0.5)	2+2 (4.0)
Co-infection IBI/malaria, n (%)	8 (1.1)	1 (6.7)	1 (0.5)	3 (1.5)	3 (1.5)	

*IBI  =  invasive bacterial infection, m  =  months*.

*Including those with IBI co-infection, ^§^Including those with malaria co-infection.

Parasite density was not significantly different for those fulfilling criteria of severe malaria (median 38,319/µl, range 25–702,500) and those not (48,544/µl, range 129–235,980, *p* = 0.799), neither when stratified by prior antimalarial treatment.

Of note, a positive malaria RDT was observed in 137 children with negative microscopy, among which 106 (77.4%) had at least one clinical or laboratory sign of severe malaria. Of them, 69.8% (74/106) reported previous antimalarial treatment, significantly higher compared to children with microscopically confirmed severe malaria (36.6%, 107/292, *p*<0.001).

### Invasive bacterial infections

Bacteremia was found in 63 (8.9%) children (Table 2), while contaminants were grown in another 27 (3.7%) samples collected. In one blood culture, two pathogens were isolated resulting in a total of 64 clinically significant isolates. Proportions of bacteremia were highest among neonates (20.0%) and children ≥5 years of age (15.8%). Pathogens most frequently isolated were non-typhoid *Salmonella* spp. (NTS, 32.8%), *Salmonella* Typhi (18.8%) *Streptococcus pneumoniae* (18.8%) and *Escherichia coli* (12.5%) (Table 3). NTS comprised *Salmonella* Typhimurium (n = 12), *Salmonella* Enteritidis (n = 8) and *Salmonella* Telelkibir (n = 1). *E. coli*, NTS and *Salmonella* Typhi were the most frequently isolated pathogens among children <1 year of age, 1–5 years and ≥5 years of age respectively (Table 3).

**Table 3:** Clinically significant organisms isolated from blood culture and age-specific frequencies.

	All	<1 m	1–11 m	12–23 m	24–59 m	≥60 m	Age in months, median (IQR)
All pathogenic bacteria	64	3	14	18	13	16	21 (9–54)
Non-typhoid *Salmonella*	21 (32.8)		5 (35.7)	8 (44.4)	6 (46.2)	2 (12.5)	19 (10–36)
*Salmonella* Typhi	12 (18.8)			1 (5.6)	3 (23.1)	8 (50.0)	75.5 (45–114)
*Streptococcus pneumoniae*	12 (18.8)	1 (33.3)	3 (21.4)	5 (27.8)	2 (15.4)	1 (6.3)	12.5 (8.5–25)
*Escherichia coli*	8 (12.5)	1 (33.3)	5 (35.7)		1 (7.7)	1 (6.3)*	7.5 (4–16.5)
*Staphylococcus aureus*	3 (4.7)		1 (7.1)			1 (6.3)	
*Shigella spp.*	2 (3.1)			1 (5.6)	1 (7.7)	1 (6.3)	
*Neisseria meningitidis*	2 (3.1)			1 (5.6)		1 (6.3)	
*Haemophilus influenzae*	1 (1.6)					1 (6.3)	
*Streptococcus pyogenes*	1 (1.6)	1 (33.3)					
*Aerococcus viridans*	1 (1.6)			1 (5.6)			
*Leuconostoc*	1 (1.6)					1 (6.3)*	

*Data displayed are numbers and percentages of clinically significant bacteria isolated*.

*m =  months, IQR  =  interquartile range*.

*In one patient 2 pathogens were isolated (E. coli and Leuconostoc).

The median (interquartile range) blood volume per blood culture vial sampled was 0.8 ml (0.3–1.2) and 2.1 ml (1.1–3.0) for children <2 months and ≥2 months of age respectively. In 15/170 (8.8%) and 10/170 (5.9%) children blood culture bottles were under-filled (<0.5 ml) and overfilled (>5.0 ml) respectively.

Bacterial meningitis was confirmed in 6/19 (31.6%) children for whom lumbar puncture was performed; pathogens were *Streptococcus pneumoniae* (n = 3), *Neisseria meningitidis* W135 (n = 1), *Haemophilus influenzae* (n = 1) and *Klebsiella pneumoniae* (n = 1). There were five additional children with probable meningitis who had *S. pneumoniae* (n = 4) and *N. meningitidis* W135 (n = 1) isolated from blood cultures.

### Co-infections of severe malaria and invasive bacterial disease

**Figure 1:**
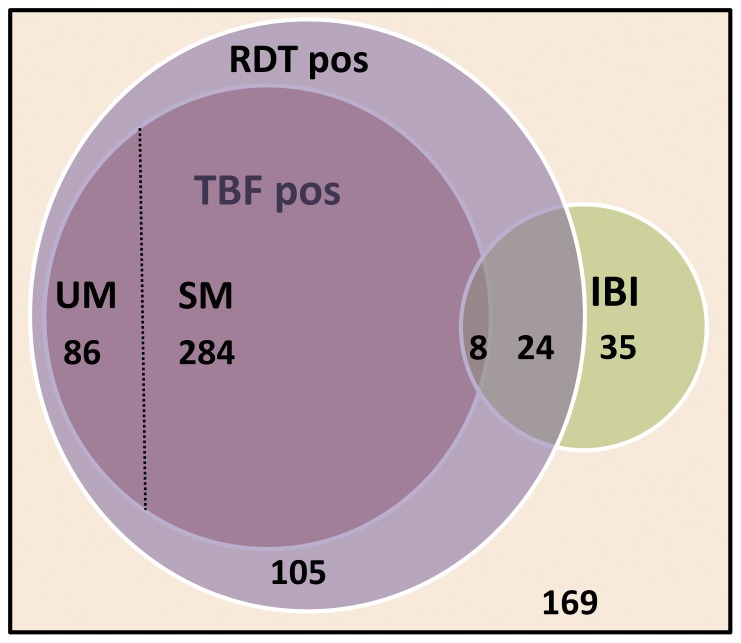
Numbers of children with positive microscopy, RDT and IBI. *TBF, RDT and IBI results for all children (n = 711), 169 children tested negative on all three items. Areas in Venn-diagram approximately to scale. TBF  =  thick blood film, RDT  =  rapid diagnostic test, IBI  =  invasive bacterial infection, SM  =  severe malaria, UM  =  uncomplicated malaria*.

Distribution of positive microscopy, RDT and IBI among children included are displayed in Figure 1. *P. falciparum* parasites were present in 7/63 (11.1%) and 1/6 (16.7%) children with bacteremia and meningitis respectively. Conversely, among the children with severe malaria 8/292 (2.7%) had IBI. No significant difference in parasite densities between children with severe malaria (median 38,319/µl, range 25–702,500) and severe malaria/IBI co-infection (79,365.5/µl, range 271–338,000) was observed (*p* = 0.5).

The RDT was positive in 30/63 (47.6%) children with bacteremia and was significantly more frequent among children with NTS (17/21, 81.0%) compared to children with other pathogens isolated (13/42, 31.0%, *p*<0.001).

### Proportions of malaria and invasive bacterial infections

**Figure 2:**
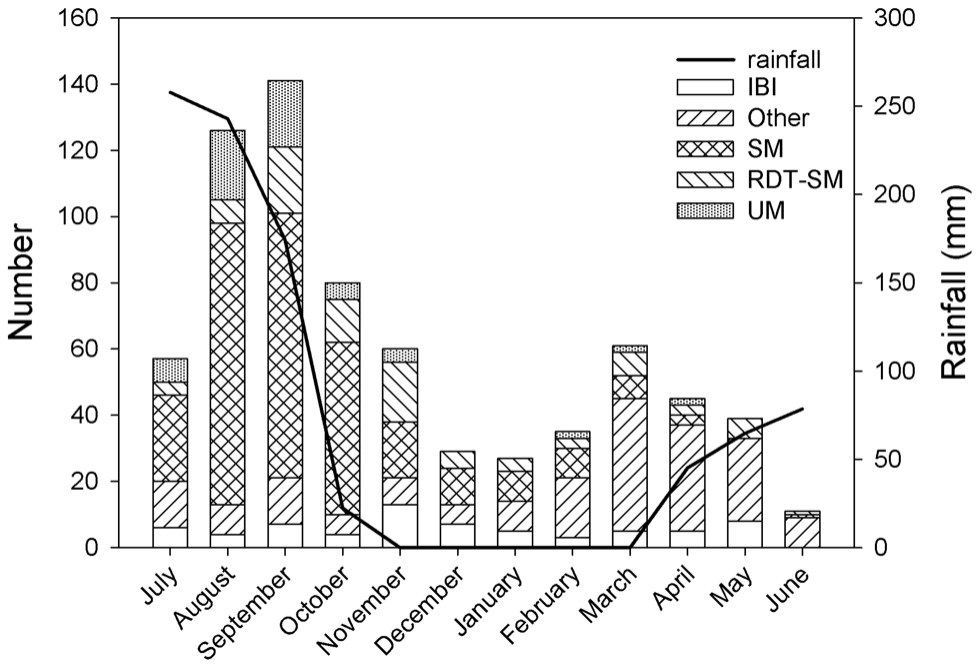
Distribution of severe malaria (SM) and invasive bacterial infection (IBI) by month. *IBI  =  invasive bacterial infection, SM  =  severe malaria, RDT-SM  =  signs of severe malaria with negative microscopy and positive RDT, UM  =  uncomplicated malaria, Other  =  all other children included not fulfilling criteria of SM or IBI*.

The proportion of severe malaria per month ranged from 0.0%–62.7% among all children included with a peak from August to October (Figure 2), while the proportion of IBI ranged from 0.0%–24.1% per month with a peak in November – January after the rainy season, mainly due to a high number of NTS infections (62.5%, 15/24 bacteremia cases), and a smaller increase in May.

### Antibiotic resistance patterns of bacterial pathogens

Table 4 displays the antibiotic resistance rates of the *Enterobacteriaceae*: 19/21 NTS isolates were MDR, of them one *Salmonella* Typhimurium was in addition ESBL producing and one *Salmonella* Enteritidis isolate showed DCS (ciprofloxacin MIC-value 0.25 µg/ml, nalidixic acid MIC-value >256 µg/ml). All other NTS isolates were susceptible to ciprofloxacin (MIC-values ≤0.023 µg/ml). *Salmonella* Telelkibir was susceptible to all antibiotics tested. Among the *Salmonella* Typhi isolates, 10/12 (83.3%) were resistant to both chloramphenicol and TMP-SMX but all were susceptible to ampicillin and ciprofloxacin (MIC-values ≤0.008 µg/ml). *Shigella flexneri* was resistant to ampicillin, chloramphenicol and TMP-SMX, *Shigella dysenteriae* was resistant to ampicillin and TMP-SMX. All NTS, *Salmonella* Typhi and *Shigella* isolates were susceptible to azithromycin (MIC-values ≤6.0, ≤2.0 and ≤1.0 µg/ml respectively). Three *E. coli* isolates were confirmed ESBL producers and co-resistant to ciprofloxacin, two of them were also co-resistant to gentamicin. No resistance to the carbapenem antibiotics tested was detected.

**Table 4:** Antibiotic resistance patterns of *Enterobacteriaceae*.

	Non-typhoid *Salmonella*	*Salmonella* Typhi	*Shigella* spp.	*Escherichia coli*	*Klebsiella pneumoniae* *
	n = 21	n = 12	n = 2	n = 8	n = 1
**Antibiotic**	**n (%) resistant isolates**				
Ampicillin	19 (90.5)	0	2	7 (87.5)	1
Chloramphenicol	19 (90.5)	10 (83.3)	1	2 (25.0)	1
TMP-SMX	19 (90.5)	10 (83.3)	2	6 (75.0)	1
MDR	19 (90.5)	0	NA	NA	NA
Nalidixic acid	1 (4.8)	0	0	NA	NA
Ciprofloxacin	NA	NA	0	5 (62.5)	0
DCS	1 (4.8)	0	NA	NA	NA
ESBL confirmed	1 (4.8)	0	0	3 (37.5)	0
Azithromycin	0	0	0	NA	NA
Gentamicin	NA	NA	NA	4 (50.0)	0
Meropenem/Ertapenem	NA	NA	NA	0	0

*DCS  =  decreased ciprofloxacin susceptibility, ESBL  =  extended-spectrum beta-lactamase, MDR  =  multidrug resistant, NA  =  non applicable, TMP-SMX  =  trimethoprim-sulfamethoxazole*.

*Isolated from CSF.

All *S. pneumoniae* isolates (n = 11, isolated from blood) were susceptible to ceftriaxone (MIC values ≤0.047 µg/ml in 10/11 and 0.19 µg/ml in the remaining one) and 10/11 were susceptible to penicillin (MIC-values ≤0.032 µg/ml in 9/10 isolates and 0.064 in one). The remaining isolate had MIC-values (0.094 µg/ml) above the CLSI meningitis susceptibility breakpoint (0.06 µg/ml). All *S. pneumoniae* isolates were susceptible to erythromycin but most 9/11 (81.8%) were resistant to TMP-SMX. The *N. meningitidis* isolates were susceptible to penicillin (MIC-values 0.047 and 0.064 µg/ml) and ceftriaxone (MIC-values 0.002 µg/ml for both isolates) as was *H. influenzae* (MIC-value 0.006 µg/ml)

All three *S. aureus* isolates were methicillin susceptible as well as susceptible to clindamycin, gentamicin and TMP-SMX; three and one of them were resistant to tetracycline and erythromycin respectively.

A total of 28.2% (205/728) children reported antibiotic use <48 h prior to sampling; most frequently administered antibiotics were TMP-SMX (35.1%) and amoxicillin or ampicillin (45.9%). Bacteria were significantly more frequently isolated among children on antibiotics (13.1%, 26/199) compared to those who reported no prior antibiotic use (7.3%, 37/510, *p* = 0.015).

### Case-fatality rates

For 14 children the final outcome could not be ascertained because they were referred to a tertiary care center (n = 8) or left the hospital before completion of treatment (n = 6). For 697 (98.0%) children hospital outcome was known: 58/697 (8.3%) children died, of them 20 (34.5%) and 15 (25.9%) were diagnosed with severe malaria and IBI respectively. CFR were significantly higher among children with IBI (23.4%) versus severe malaria (6.8%, *p*<0.001), none of the children with severe malaria/IBI co-infection (n = 8) died. Among children with a positive RDT and negative microscopy with signs of severe malaria 11/110 (10.0%) died, of them six had IBI.

CFR for IBI was highest among children <1 year of age (52.9%, 9/17). Among children with bacteremia, deaths occurred among children infected by *S. pneumoniae* (4/12), *E. coli* (4/8), NTS (3/20), *S. aureus* (2/3) and *N. meningitidis* (1/2). None of the patients with *Salmonella* Typhi bacteremia died. Among children with confirmed and probable meningitis 4/10 (40.0%) died (three *S. pneumoniae*, one *N. meningitidis*), while 8/46 (17.4%) children with cerebral malaria died.

## Discussion

The present study showed that, among children ill enough to be admitted to rural health facilities in Burkina Faso, severe malaria was the leading cause of morbidity (40% of admissions) and mortality (one third of deaths). IBI were however not uncommon and were associated with a significantly higher CFR compared to malaria. The relative proportions of severe malaria and IBI in febrile and/or severely ill children differed dramatically according to the seasons as well as the bacterial pathogens isolated, with a peak of severe malaria during the rainy season and a relative increase of IBI (mainly due to NTS) shortly thereafter. High resistance rates to first line antibiotics were observed, particularly among Gram-negative pathogens.

This study has several limitations. First, a substantial proportion of children fulfilling inclusion criteria was not included. As age and mortality rate did not differ significantly, non-inclusion probably occurred at random although the lower body temperatures may suggest less severe disease in the non-included patients. Second, blood cultures intrinsically have moderate sensitivity (*e.g*. 40–80% in the case of typhoid fever [16]), which is further lowered by antibiotic exposure prior to sampling. In the present study, the first-line antibiotics used prior to sampling (ampicillin and TMP-SMX) were mostly ineffective to the pathogens retrieved and the percentage of bacteremia was higher among children who were on antibiotics before sampling. Nevertheless, more susceptible and fragile strains, like *S. pneumoniae*, may have been missed. Quality indicators of the current blood culture system were satisfying: the percentage of contaminants (3.7%) was acceptable and lower compared to other studies performed in malaria endemic settings (8–14.3%) [2], [17]–[20]. Performance of a lumbar puncture was left to the decision of the health care worker, and the actual number of meningitis cases may have been higher as in only nineteen children with clinical signs of cerebral malaria and/or meningitis a lumbar puncture was performed. Health care workers in rural Africa may be reluctant to perform a lumbar puncture because of lack of experience, appropriate materials or microbiological facilities. In addition, the case definition of severe malaria might have been too stringent: indeed, some children may have been erroneously excluded from the diagnosis of severe malaria as no parasites were seen by microscopy, whereas the PfHRP2-detecting RDT was positive. This was supported by the significantly higher proportion of antimalarial treatment prior to admission in this group compared to microscopy positive children. The frequent use of TMP-SMX (which is an antimalarial as well [21]) prior to admission may have further contributed to negative microscopy. On the other hand, some children may have had asymptomatic carriage of *P. falciparum* parasites, which is not uncommon in Burkina Faso [22], while being ill due to a bacterial or viral infection [23], [24]. Furthermore, with the available diagnostic methods we could not establish a final diagnosis for all children included. Finally, the limited number of children for whom HIV testing was performed precluded assessment of associations with IBI and/or severe malaria.

Bacterial co-infections among children with severe malaria were less frequent (2.7%) compared to those reported from other studies (4.6%–8.3.%) [2], [17], [25]–[29] as was *P. falciparum* co-infection among children with bacteremia (11.1% versus 14.7%–21.6%) [2], [19], [30]. This may be explained by the frequent use of antimalarial treatment before admission. When considering positive RDT results, the proportion of co-infections was substantially higher (49.2% of children with bacteremia had positive RDT), and comparable to a recent study from Tanzania (56.9%) [28].

Except for surveillance of meningitis epidemics and a single study on blood culture isolates in a tertiary care urban center [31], there are no previous data about the burden of community acquired bacteremia in Burkina Faso, in particular from rural areas. The high proportion of NTS isolates is consistent with studies performed in other rural malaria endemic settings in sub-Saharan Africa [18], [19], [26]–[29], [32], although we isolated NTS mainly after instead of during the rainy season [18], [26], [27]. *Salmonella* Typhi was relatively frequent in our study compared to others performed in similar settings [2], [32], [33], possibly related to specific local environmental conditions or to the inclusion of children ≥5 years old. Although *Salmonella* Typhi is more common in older children [16], [28], it may also occur in children ≤5 [28], [34] as shown in the present study, and incidence rates of *Salmonella* Typhi bacteremia have reported to be even higher among children 2–5 years compared to children ≥5 [35], [36].

Resistance rates among community-acquired pathogens were high, particularly among the *Enterobacteriaceae*. The high resistance rates to the first line antibiotics TMP-SMX and amoxicillin/ampicillin requires extension of surveillance studies to other populations (*e.g*. adults, outpatients) and places in the country and revision of the current national treatment guidelines. Of concern are the findings of DCS and ESBL in one NTS isolate each and especially the presence of ESBL with co-resistances to gentamicin and fluoroquinolone antibiotics among *E. coli*, precluding treatment options with commonly available antibiotics. All *S. pneumoniae, N. meningitidis* and *H. influenzae* isolates were susceptible to ceftriaxone, which is the recommended treatment for bacterial meningitis in Burkina Faso.

CFR for IBI were significantly higher compared to severe malaria (23.4% and 6.8% respectively) and existence of IBI as well as appropriate case management should be more emphasized at all levels of health care.

As malaria may predispose to NTS bacteremia [37], [38], reduction of malaria transmission would not only lead to a reduction in morbidity and mortality due to malaria but could also reduce the burden of NTS bacteremia [38]. As main reservoirs for human invasive NTS have to date not been explored extensively [39], containment of risk factors (malaria, HIV, malnutrition [39]) appears the best preventive measure. Introduction of the 13-polyvalent pneumococcal vaccine and vaccination against typhoid fever as well as improved hygiene could further reduce the burden of community-acquired childhood bacteremia in this area.

In conclusion, although severe malaria was the main cause of disease among children admitted to a rural hospital in Burkina Faso, invasive bacterial infections were not uncommon and had higher CFR. The high proportion and CFR of community acquired bacteremia as well as the high antibiotic resistance rates of the pathogens require improvement in hygiene, better diagnostic methods and emphasize the need of blood culture surveillance and revision of current treatment guidelines.
